# Association between Systolic Blood Pressure Variability and Incident Aortic Stenosis

**DOI:** 10.3390/jcm13133881

**Published:** 2024-07-01

**Authors:** Hyun-Jung Kim, Ji-Eon Kim, Jae-Seung Jung, Hee-Jung Kim, Ho-Sung Son

**Affiliations:** 1 Department of Preventive Medicine, College of Medicine, Korea University, Seoul 02841, Republic of Korea; moole02@naver.com; 2 Department of Thoracic and Cardiovascular Surgery, Korea University Anam Hospital, Korea University, Seoul 02841, Republic of Korea; jieonkim82@gmail.com (J.-E.K.); heartistcs@korea.ac.kr (J.-S.J.)

**Keywords:** aortic valve stenosis, blood pressure variability, big data

## Abstract

**Background**: This study investigated the potential link between blood pressure variability (BPV) and the incidence of aortic stenosis (AS) using Korean National Health Insurance Service data from 2002 to 2019. **Methods**: We collected annual systolic blood pressure variability (SBPV) measurements consisting of three consecutive blood pressure readings each year over three years. The obtained SBPV data was divided into five quantiles, with the highest quintile representing a high fluctuation of blood pressure. **Results**: Analyzing 9,341,629 individuals with a mean age of 40.7 years, the study found 3981 new AS diagnoses during an average 8.66-year follow-up. Independent predictors for AS included higher blood pressure levels and elevated systolic blood pressure variability (SBPV). The hazard ratios (HR) for different SBPV quintiles compared to the reference (1st quintile) were as follows: 2nd quintile HR 1.09 (*p* = 0.18), 3rd quintile HR 1.13 (*p* = 0.04), 4th quintile HR 1.13 (*p* = 0.04), and 5th quintile HR 1.39 (*p* < 0.001). **Conclusion**: Our findings suggest that both hypertension and high fluctuations in SBP during consecutive visits are associated with an increased risk of incident AS. These results emphasize the importance of blood pressure management and stability in the prevention of AS.

## 1. Introduction

Aortic stenosis (AS) is a common valvular heart disease among the elderly, with its prevalence increasing with advancing age. At the age of 65, AS is observed in approximately 5% of individuals, and as the population ages, the prevalence of AS is sharply rising [1]. It is crucial to understand the risk factors associated with incident AS and to investigate modifiable health conditions in order to reduce future medical burdens.

Previous epidemiological studies have demonstrated an association between hypertension and a higher incidence of AS [2]. A large longitudinal cohort study using data from the United Kingdom database revealed that higher systolic, diastolic, and pulse pressures were linked to an increased risk of AS compared to individuals with normal blood pressure (BP) [3]. Hypertension plays a significant role in initiating and accelerating the development of AS.

With advancements in our knowledge of hypertension pathology, blood pressure variability (BPV), which is one of the indices of BP, has emerged as a prominent predictor of cerebrocardiovascular events [4,5,6]. Prior research suggests that fluctuations in blood pressure can directly damage end organs or indicate underlying conditions such as aging, arterial stiffness, or response to antihypertensive medication. Therefore, high blood pressure variability is strongly associated with a higher incidence of adverse events in individuals. Monitoring the variation in BP across multiple visits provides a reliable prediction for adverse events beyond the usually measured blood pressure (systolic or diastolic BP) in relation to mortality, cardiac, cerebral, and vascular diseases.

Despite the notable clinical implications of BPV on cardiovascular events, the potential association between BPV and incident AS has not been studied yet. Therefore, in this study, we aimed to investigate the feasibility of long-term systolic blood pressure variability (SBPV) as an independent predictor for incident AS. We utilized national cohort longitudinal data linked with consecutive BP measurements obtained during health screening examinations.

## 2. Materials and Methods

### 2.1. Data Source

This nationwide retrospective cohort study utilized the Korean National Health Insurance (NHI) claims database as the primary data source. The NHI system provides coverage for over 97% of the entire Korean population, which amounts to more than 50 million individuals [7]. The NHI database encompasses comprehensive information regarding medical service utilization throughout Korea. It includes data on diagnoses based on the International Classification of Diseases Tenth Revision (ICD-10) codes, both inpatient and outpatient claims data, primary and secondary diagnosis codes, as well as treatments.

For this study, the NHI database was linked with the National Health screening data provided by the NHI. The National Health screening data consist of a health checkup database covering the entire Korean population. As part of the national health screening initiative, all insured adults are eligible to participate in the health screening program and are recommended to undergo standardized medical examinations every one or two years.

By leveraging the combined data from the NHI claims database and the National Health screening data, we were able to conduct a comprehensive analysis to investigate the association between long-term SBPV and incident AS in this cohort study.

### 2.2. Study Population and Outcome of Interest

The primary outcome of interest in this study was the first diagnosis of aortic stenosis (AS) during the follow-up period. 

From the Korean NHI claims database, we identified subjects who underwent the National Health screening program from 2002 to 2019 and had three consecutive annual visits. The study population included individuals aged 20 years or older. Those who were diagnosed with aortic stenosis were defined as individuals who had visited the clinic at least twice with an ICD code of I350. To ensure the accuracy of the newly diagnosed AS cases, a three-year washout period was applied.

**Figure 1:**
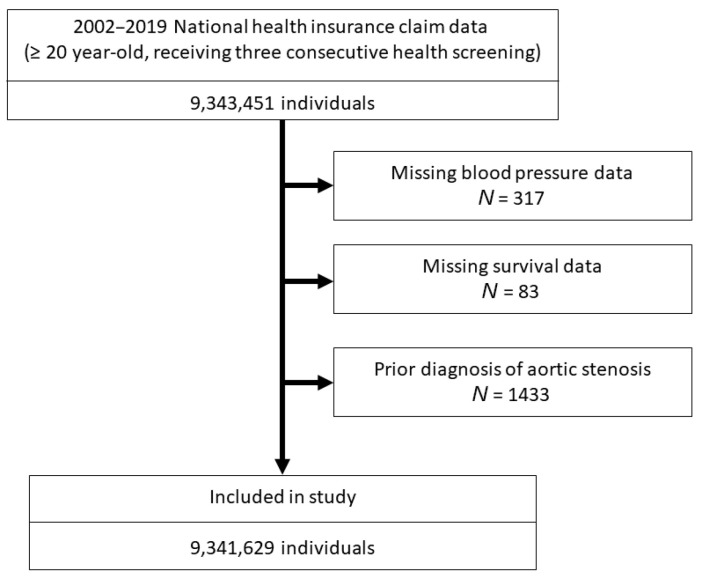
Flow chart of study cohort.

Initially, a total of 9,343,451 subjects were identified in the national health insurance database as meeting the inclusion criteria. However, certain exclusions were applied. Subjects with missing data regarding diastolic pressure (*N* = 317) and survival (*N* = 83), as well as those who had a prior diagnosis of aortic stenosis before the indexed date (*N* = 1422), were excluded from the analysis. Finally, a total of 9,341,629 subjects were included in this study (Figure 1).

#### Definitions of Blood Pressure Grouping and Variability Estimation

Participants were categorized into five groups by BP level, as follows:

1. Normal, SBP < 120 mmHg and DBP < 80 mmHg
2. Prehypertension 1, 120 mmHg ≤ SBP < 130 mmHg and DBP < 80 mmHg
3. Prehypertension 2, 130 mmHg ≤ SBP < 140 mmHg and 80 mmHg ≤ DBP < 90 mmHg
4. Hypertension 1, 140 mmHg ≤ SBP < 180 mmHg and 90 mmHg ≤ DBP < 120 mmHg
5. Hypertension 2, 180 mmHg ≤ SBP and 120 mmHg ≤ DBP.

To estimate SBPV, systolic BP measurements were obtained from three consecutive health screening exams conducted annually. During the health exams, an individual’s BP was measured using authorized devices and monitored by trained nurses. The SBPV was calculated based on the three consecutive systolic BP values obtained from the health screening exams.

The SBPV was divided into quintiles, and a high BPV was defined as the 5th quintile, representing the highest variability in blood pressure.

BPV was calculated by average real variability (ARV). ARV is the average of the absolute differences between consecutive values [5]. These measures were used to assess the variability in systolic blood pressure across the three consecutive health screening exams.

### 2.3. Aortic Stenosis Risk Profiles

Cardiovascular risk profiles were obtained from the latest health screening exam (last exam) among the three consecutive exams conducted. The following parameters were obtained: blood pressure (systolic and diastolic), fasting blood glucose, total cholesterol, body mass index (BMI), and smoking status. The presence of chronic kidney disease (CKD) was investigated through proteinuria (urine stick test), history of CKD with calculated glomerular filtration rate (GFR), renal replacement therapy, and history of kidney transplantation. The definition of variables based on ICD codes is summarized in Supplementary Table S1.

### 2.4. Validating Diagnosis of Aortic Stenosis

To validate the algorithm used for detecting AS diagnosis in the NHI database, medical records, including echocardiography data, of patients diagnosed with ICD code I350 (AS) at Korea University Anam Hospital were examined. In this study, AS was defined as valve hemodynamics with a peak aortic valve velocity of ≥2 m/s or mean aortic valve gradient ≥20 mmHg, following the recommendations of the American Heart Association/American College of Cardiology. Between 2015 and 2018, a total of 200 patients were reviewed. Among those who received an outpatient visit with an ICD code of I350 and visited the clinic more than two times, 190 subjects met the diagnostic criteria for aortic stenosis (positive predictive value, 95%; negative predictive value, 100%).

### 2.5. Statistical Analysis

The study aimed to investigate the incidence of AS in Korea between 2002 and 2019. Incident cases were defined as newly registered patients with AS in the NHIS during a given year. The variability of SBP (systolic blood pressure) was divided into five quintiles, with the 5th quintile representing the highest variability. Baseline characteristics were described by the five quintiles, including stratified sex (male and female), age (<60, 60≤), BP (five grades), ARV (quintiles), fasting blood sugar (FBS) levels (<100, 100≤ and <126, 126≤), cholesterol levels (<200, 200≤ and <240, 240≤), body mass index (BMI) (18.5<, 18.5≤ and <25.0, 25.0≤), proteinuria levels (<3+, 3+≤), smoking status (non-smoker, <20 pack/year, 20 pack/year≤), and chronic kidney disease (CKD) status (no CKD, history of CKD, on dialysis, kidney transplantation). Missing data were not included in the table, and all variables are presented as frequencies and percentages (Table 1).

For each study participant, person-years were calculated from the index date of diagnosis to the corresponding end of follow-up. Person-years were contributed by study participants only while they were at risk (i.e., alive and living in Korea without a diagnosis of AS). The incidence rate was calculated by dividing the number of incident cases by 100,000 total person-years.

Cox proportional hazard models were utilized for analyzing the relationship between incident AS and covariates in both univariable and multivariable analyses. The multivariable analysis for incident AS was adjusted for covariates such as age, sex, five blood pressure grades, quintiles of ARV, BMI, cholesterol levels, fasting glucose levels, proteinuria levels, smoking status, and chronic kidney disease status (CKD, on dialysis, kidney transplantation) (Table 2 and Table 3, Supplementary Table S2).

**Figure 2:**
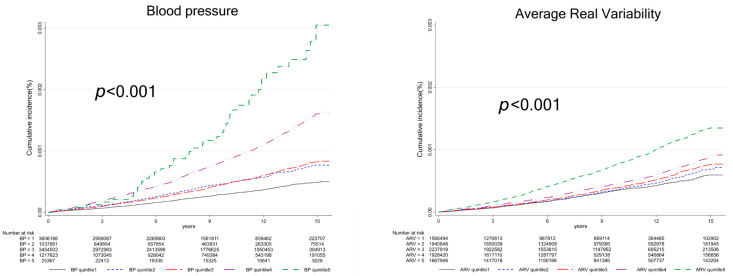
Cumulative incidence of aortic stenosis according to grade of blood pressure, and quintiles of systolic blood pressure variability (Average real variability (ARV)). BP 1, SBP < 120 mmHg and DBP < 80 mmHg; BP 2, 120 mmHg ≤ SBP < 130 mmHg and DBP < 80 mmHg; BP 3, 130 mmHg ≤ SBP < 140 mmHg and 80 mmHg ≤ DBP < 90 mmHg; BP 4, 140 mmHg ≤ SBP < 180 mmHg and 90 mmHg ≤ DBP < 120 mmHg; BP 5, 180 mmHg ≤ SBP and 120 mmHg ≤ DBP.

The plots depicting incident AS according to the five blood pressure levels and the quintiles of ARV were estimated using the Kaplan–Meier method and assessed with the log-rank test (Figure 2).

All statistical analyses were performed using SAS version 9.3 (SAS Institute, Cary, NC, USA), and a two-tailed *p*-value of less than 0.05 was considered statistically significant.

### 2.6. Ethics Approval

The present study protocol was reviewed and approved by the Institutional Re-view Board of Korea University Anam Hospital (No. 2019AN0329, Date: 25 July 2019). Informed consent was waived by the IRB’s decision.

## 3. Results

### 3.1. Study Cohort

The study cohort had a mean age of 40.7 ± 12.1 years, and 35.4% of the participants were female. The average follow-up duration was 8.66 ± 4.6 years. A total of 3981 individuals were diagnosed with AS during the study period. The incidence rate of AS was calculated at 42.6 per 100,000 person-years. Higher SBPV quintiles based on the ARV method were associated with older age, male gender, smoking, higher BP levels, BMI, cholesterol levels, fasting blood glucose levels, proteinuria, and a higher prevalence of CKD (Table 1). The incidence rate of AS was positively correlated with increasing ARV and blood pressure grades. Older age, higher BMI, cholesterol levels, fasting glucose levels, proteinuria, heavy smoking, and presence of CKD were associated with an increased incidence rate of AS (Table 2).

Abbreviations: Q, quintile; ARV, average real variability; BP, blood pressure; FBS, fasting blood sugar; BMI, body mass index; CKD, chronic kidney disease; TPL, transplantation BP definition: normal, SBP < 120 mmHg and DBP < 80 mmHg; prehypertension 1, 120 mmHg ≤ SBP< 130 mmHg and DBP < 80 mmHg; prehypertension 2, 130 mmHg ≤ SBP < 140 mmHg and 80 mmHg ≤ DBP < 90 mmHg; hypertension 1, 140 mmHg ≤ SBP < 180 mmHg and 90 mmHg ≤ DBP < 120 mmHg; hypertension 2, 180 mmHg ≤ SBP and 120 mmHg ≤ DBP.

### 3.2. Association of BP Grades and Systolic Blood Pressure Variability with Incident Aortic Stenosis

Both higher blood pressure grades and high SBPV were associated with an increased risk of incident AS. Higher blood pressure grades were significantly associated with a higher incidence rate of AS compared to the reference grade. The incidence rates for blood pressure grades were 2.98, 4.75, 4.84, 9.55, and 16.58 per 100,000 person-years (Table 3). The adjusted hazard ratios and 95% confidence intervals for each grade compared to the first grade were 1.18 (1.05–1.33), 1.12 (1.02–1.21), 1.33 (1.21–1.46), and 1.52 (1.11–2.08), respectively (all *p* < 0.05) (Table 3). High SBPV (5th quintile group) based on ARV was significantly associated with a higher incidence rate of AS compared to the reference (1st quintile group). Increasing quintiles of SBPV were associated with a proportional increase in the incidence rate. The incidence rates for ARV quintiles from 1st to 5th were 3.46, 3.94, 4.31, 5.00, and 8.06 per 100,000 person-years (Table 3). The adjusted hazard ratio and 95% confidence interval for the 5th quintile group were 1.39 (1.24–1.55) compared to the reference (*p* < 0.001) (Table 3). The cumulative incidence rate of AS by blood pressure grades and SBPV is shown in Figure 2.

## 4. Discussion

To the best of our knowledge, this study is the first investigation to explore the association between SBPV and incident AS. In this longitudinal national cohort study with a mean follow-up of 9 years, we found that higher blood pressure grades and high SBPV (5th quintile) were associated with an increased risk of incident AS. 

Hypertension is a well-established independent risk factor for incident AS. Previous cohort studies have identified several risk factors for incident AS, including age, hypertension, diabetes, hypercholesterolemia, and smoking. These risk factors are similar to those associated with atherosclerosis, suggesting a shared mechanism between incident AS and atherosclerosis. The exact contribution of hypertension to the development of incident AS is not fully understood. However, studies have shown that hypertension is an independent risk factor compared to other cardiovascular risk factors such as diabetes and dyslipidemia [8]. The mechanical stress imposed by high blood pressure is believed to play a role in the initiation and progression of AS. The pathophysiological process of AS begins with endothelial dysfunction caused by mechanical injury, leading to lipid deposition and inflammation. Subsequent mechanical stress contributes to the progression of AS. High blood pressure may influence all phases of AS development, from initiation to propagation. Prospective and experimental studies have suggested that hypertension may be a leading factor in the incidence and progression of AS [9]. 

The mechanism underlying the association between BPV and incident AS has not been fully elucidated. Several hypotheses have been proposed to explain the causality of BPV in the development of cardiovascular disease. BPV has been shown to be influenced by factors such as aging, arterial compliance, and individual responses to anti-hypertensive drugs. Although the pathophysiology is unclear, evidence suggests that BPV is correlated with arterial stiffness and renal function. Long-term BPV has been positively associated with carotid intima-media thickness in a 20-year follow-up study [10]. Moreover, BPV not only serves as a predictor but also as a potential treatment target. Previous studies have shown that reducing BPV is associated with improved clinical outcomes, beyond the reduction in mean blood pressure. Specific anti-hypertensive drugs may effectively reduce BPV and impact clinical outcomes [11,12]. For example, a study demonstrated that amlodipine effectively reduced BPV compared to atenolol or lisinopril [13]. These findings suggest that targeting BPV reduction with specific medications may influence clinical outcomes.

Fluctuations in blood pressure over time can vary significantly among individuals, exhibiting different patterns. Large cohort studies have consistently shown that high BPV is a significant determinant of adverse cerebrovascular events beyond the usual measurements of blood pressure [4,5,14,15]. These studies have demonstrated that increased BPV is associated with a higher risk of myocardial infarction, stroke, and end-stage renal disease. Gasmanova and colleagues found that higher BPV was linked to increased hazards for all-cause mortality, coronary heart disease, stroke, and end-stage renal disease among a cohort of 2.9 million veterans [4]. Our study results support these findings by demonstrating that high SBPV is an independent risk factor for incident AS, even after adjusting for usual blood pressure.

However, there are ongoing debates regarding the role of BPV as a predictor of cardiovascular disease. One argument is that BPV may simply serve as a marker of severe atherosclerosis or arterial stiffness [16]. The causality of BPV in the development of atherosclerosis or AS has not been fully evaluated, and there are limitations in interpreting the predictive value of BPV in the context of developing AS. Second, there have been studies that did not find a significant association between BPV and adverse clinical outcomes. For instance, Mancia et al. demonstrated that BPV had no predictive value compared to mean blood pressure [17]. Third, studies focused on BPV may be subject to biases related to proper blood pressure measurement, including factors such as the device used, measurement techniques, timing, and interval between measurements. Recent papers suggest that controlling systolic blood pressure variability (BPV) may reduce cardiovascular events [18]. Thus, further research is needed to evaluate appropriate methods for obtaining BPV and to address these controversies. 

### Limitation

Because of the retrospective study design, uncontrolled confounders and selection biases may be present. First, the causality between BPV and incident AS was not clear. Our study had limited information regarding the mechanism of driving AS. There is a controversial issue regarding whether BPV is a consequence of the progression of the pathology. Therefore, the issue regarding causality cannot be excluded. However, we demonstrated the proportionally increased IR according to higher SBPV and the consistent effect of SBPV adjusted with usual blood pressure and among normotensive patients. Second, the incidence of AS was found using the diagnosis of AS based on claim data from national health insurance. Therefore, some missing or oversizing the number of diagnoses for AS could emerge. Therefore, we validated the appropriateness of our algorithm to find AS. Based on the retrospective review of the hospital database (other than the study cohort), our algorithm is acceptable, with a positive predictive value of 95%. Regarding the categorization of variability, we acknowledge that the precise values were not obtained.

## 5. Conclusions

Our study demonstrated that high BP and SBP fluctuations were associated with an increased risk of incident AS. Therefore, the BP and SBPV are reliable predictors of incident AS together. 

## Figures and Tables

**Table 1:** Baseline characteristics according to systolic blood pressure variability with categorized by five quintiles with average real variability (ARV).

Variables	ARV				
Quartiles	1Q (1,566,494)	2Q (1,940,848)	3Q (2,237,919)	4Q (1,928,420)	5Q (1,667,948)
Sex					
Female	591,509 (37.76)	697,252 (35.93)	773,325 (34.56)	674,123 (34.96)	566,541 (33.97)
Age					
Age, ~59 y	1,461,038 (93.27)	1,813,110 (93.42)	2,079,723 (92.93)	1,751,272 (90.81)	1,449,874 (86.93)
Age, 60 y ~	105,456 (6.73)	127,738 (6.58)	158,196 (7.07)	177,148 (9.19)	218,074 (13.07)
Body profiles					
Height (cm)	166.83 ± 8.65	166.83 ± 8.60	166.86 ± 8.62	166.53 ± 8.72	165.93 ± 8.82
Weight (kg)	65.66 ± 12.43	65.64 ± 12.25	65.91 ± 12.29	66.08 ± 12.37	66.39 ± 12.52
BMI					
<18.5	71,144 (4.54)	87,332 (4.5)	95,809 (4.28)	75,858 (3.93)	56,948 (3.41)
18.5≤ and <25.0	1,023,953 (65.37)	1,269,988 (65.43)	1,446,843 (64.65)	1,221,842 (63.36)	1,011,042 (60.62)
25.0≤	471,182 (30.08)	583,228 (30.05)	694,870 (31.05)	630,398 (32.69)	599,692 (35.95)
BP					
BP, normal	686,318 (43.81)	761,436 (39.23)	870,144 (38.88)	733,345 (38.03)	554,943 (33.27)
BP, pre-hypertension 1	192,537 (12.29)	236,961 (12.21)	245,515 (10.97)	209,641 (10.87)	153,297 (9.19)
BP, pre-hypertension 2	578,537 (36.93)	744,720 (38.37)	851,120 (38.03)	698,224 (36.21)	581,901 (34.89)
BP, Hypertension 1	108,714 (6.94)	196,704 (10.13)	269,067 (12.02)	283,462 (14.7)	359,676 (21.56)
BP, Hypertension 2	388 (0.02)	1027 (0.05)	2073 (0.09)	3748 (0.19)	18,131 (1.09)
Mean BP					
Systolic BP (mmHg)	119.81 ±11.41	119.76 ± 12.17	120.56 ± 13.37	121.85 ± 14.90	125.13 ± 17.92
Diastolic BP (mmHg)	75.15 ± 8.77	75.52 ± 9.09	75.92 ± 9.49	76.37 ± 10.19	78.21 ± 11.53
Pulse pressure					
<40	239,227 (15.27)	294,664 (15.18)	348,895(15.59)	323,430 (16.77)	261,455 (15.68)
4 0≤ and <60	1,252,969 (79.99)	1,536,239 (79.15)	1,729,491 (77.28)	1,422,420 (73.76)	1,164,795 (69.83)
60≤	74,298 (4.74)	109,945(5.66)	159,533 (7.13)	182,570 (9.47)	241,698 (14.49)
FBS, mg/dL					
<100	1,184,281 (75.6)	1,468,781 (75.68)	1,675,492 (74.87)	1,407,070 (72.96)	1,151,699 (69.05)
100≤ and <126	323,170 (20.63)	395,765 (20.39)	468,274 (20.92)	427,346 (22.16)	407,045 (24.4)
126≤	58,860 (3.76)	76,038 (3.92)	93,885 (4.2)	93,765 (4.86)	108,947 (6.53)
Cholesterol, mg/dL					
<200	946,189 (60.4)	1,184,685 (61.04)	1,358,281 (60.69)	1,142,563 (59.25)	961,475 (57.64)
200≤ and <240	410,479 (26.2)	516,754 (26.63)	605,138 (27.04)	528,810 (27.42)	475,316 (28.5)
240≤	126,546 (8.08)	160,161 (8.25)	190,542 (8.51)	173,930 (9.02)	167,157 (10.02)
Proteinuria					
<3+	1,539,524 (98.28)	1,908,895 (98.35)	2,199,785 (98.3)	1,892,230 (98.12)	1,628,937 (97.66)
3+≤	22,779 (1.45)	27,282 (1.41)	32,951 (1.47)	31,735 (1.65)	35,122 (2.11)
Smoking					
Non-smoker	848,100 (54.14)	1,058,080 (54.52)	1,205,062 (53.85)	1,028,067 (53.31)	890,988 (53.42)
<20 pack/year	472,074 (30.14)	597,537 (30.79)	704,159 (31.46)	588,830 (30.53)	489,414 (29.34)
20 pack/year ≤	120,320 (7.68)	156,616 (8.07)	188,257 (8.41)	176,449 (9.15)	178,562 (10.71)
CKD					
No CKD	1,561,882 (99.69)	1,934,441 (99.66)	2,229,777 (99.62)	1,919,785 (99.54)	1,655,850 (99.25)
History of CKD	4248 (0.27)	5945 (0.31)	7521 (0.34)	7967 (0.41)	10,937 (0.66)
On dialysis	411 (0.03)	543 (0.03)	729 (0.03)	797 (0.04)	1240 (0.07)
Kidney TPL	135 (0.01)	172 (0.01)	184 (0.01)	197 (0.01)	290 (0.02)

All variables showed statistically significant differences among the quintiles (*p* < 0.001).

**Table 2:** Incidence rate and univariable analysis of risk factors for incident aortic stenosis.

Variables	IR	Un Adjusted HR	95% CI	*p*
Sex				
Sex (male)	5.21	Reference (1.0)		
Sex (female)	4.29	0.69	0.65–0.74	<0.001
Age				
Age <40 y	0.74	Reference (1.0)		
40≤ and <60	5.79	7.78	6.90–8.77	<0.001
60≤ and <80	32.26	48.53	43.00–54.77	<0.001
80 ≤	140.92	249.90	169.64–368.15	<0.001
BMI				
<18.5	2.46	Reference (1.0)		
18.5≤ and <25.0	4.31	0.58	0.46–0.72	<0.001
25.0≤	6.54	1.53	1.44–1.63	<0.001
BP				
BP, normal	2.98	Reference (1.0)		
BP, Pre-hypertension 1	4.75	1.58	1.40–1.78	<0.001
BP, pre-hypertension 2	4.84	1.58	1.46–1.72	<0.001
BP, hypertension 1	9.55	3.01	2.75–3.28	<0.001
BP, hypertension 2	16.58	5.25	3.85–7.16	<0.001
FBS, mg/dL				
<100	4.03	Reference (1.0)		
100≤ and <126	6.74	1.69	1.57–1.81	<0.001
126≤	11.71	2.95	2.65–3.27	<0.001
Cholesterol, mg/dL				
<200	4.34	Reference (1.0)		
200≤ and <240	5.66	1.31	1.22–1.40	<0.001
240≤	6.79	1.58	1.43–1.74	<0.001
Proteinuria				
<3+	4.77	Reference (1.0)		
3+≤	14.75	3.18	2.73–3.71	<0.001
Smoking				
Non-smoker	5.22	Reference (1.0)		
<20 pack/year	3.00	0.58	0.53–0.63	<0.001
20 pack/year ≤	9.72	1.87	1.72–2.03	<0.001
CKD				
No CKD	4.71	Reference (1.0)		
History of CKD	54.43	11.79	10.07–13.80	<0.001
On dialysis	84.03	19.57	12.47–30.72	<0.001
Kidney TPL	38.17	9.21	2.30–36.85	<0.001

Incidence rate: 100,000 person-year. Abbreviations: BP, blood pressure; FBS, fasting blood sugar; BMI, body mass index; CKD, chronic kidney disease; TPL, transplantation; IR, incidence rate; CI, confidential interval BP definition: normal, SBP < 120 mmHg and DBP < 80 mmHg; prehypertension 1, 120 mmHg ≤ SBP< 130 mmHg and DBP < 80 mmHg; prehypertension 2, 130 mmHg ≤ SBP < 140 mmHg and 80 mmHg ≤ DBP < 90 mmHg; hypertension 1, 140 mmHg ≤ SBP < 180 mmHg and 90 mmHg ≤ DBP < 120 mmHg; hypertension 2, 180 mmHg ≤ SBP and 120 mmHg ≤ DBP.

**Table 3:** Multivariable analysis for incidence rate, hazard ratio, and 95% confidential interval for incident aortic stenosis by blood pressure level and quintile (Q) of ARV.

Variables	IR	Adjusted HR	95% CI	*p*
Blood pressure				
Normal	2.98	Reference (1.0)		
Pre-hypertension 1	4.75	1.18	1.05–1.33	0.01
Pre-hypertension 2	4.84	1.12	1.02–1.21	0.01
Hypertension 1	9.55	1.33	1.21–1.46	<0.001
Hypertension 2	16.58	1.52	1.11–2.08	0.01
ARV				
1Q	3.46	Reference (1.0)		
2Q	3.94	1.09	0.96–1.23	0.18
3Q	4.31	1.13	1.01–1.27	0.04
4Q	5.00	1.13	1.01–1.27	0.04
5Q	8.06	1.39	1.24–1.55	<0.001
Cholesterol, mg/dL				
<200		Reference (1.0)		
200≤ and <240	1.02	1.02	0.95–1.09	0.59
240≤	1.05	1.05	0.96–1.16	0.33

Adjusted with age, female, BMI, cholesterol, fasting glucose, proteinuria, smoking, and chronic kidney disease. Abbreviations: Q, quintile; ARV, average real variability, HR, hazard ratio; CI, confidential interval. Incidence rate: 100,000 person-years. BP definition: normal, SBP < 120 mmHg and DBP < 80 mmHg; prehypertension 1, 120 mmHg ≤ SBP< 130 mmHg and DBP < 80 mmHg; prehypertension 2, 130 mmHg ≤ SBP < 140 mmHg and 80 mmHg ≤ DBP < 90 mmHg; hypertension 1, 140 mmHg ≤ SBP < 180 mmHg and 90 mmHg ≤ DBP < 120 mmHg; hypertension 2, 180 mmHg ≤ SBP and 120 mmHg ≤ DBP.

## Data Availability

Restrictions apply to the availability of these data. Data were obtained from Korea National Health Insurance Service and are available at https://nhiss.nhis.or.kr/bd/ay/bdaya001iv.do (accessed on 17 June 2024) with the permission of Korea National Health Insurance Service.

## Supplementary Materials

The following supporting information can be downloaded at: https://www.mdpi.com/article/10.3390/jcm13133881/s1, Table S1: Definitions of comorbidity and diagnosis. Table S2: Multivariable analysis for the incident aortic stenosis.
