# Supplementary material for: Association between Systolic Blood Pressure Variability and Incident Aortic Stenosis
Source: J Clin Med. 2024 Jul 1;13(13):3881. doi: 10.3390/jcm13133881 (PMC11242494; doi:10.3390/jcm13133881)
Supplement: Supplementary file 1 [file jcm-13-03881-s001.zip › jcm-3054280-supplementary.pdf]

Supplementary Table S1. Definitions of comorbidity and diagnosis

Variables	ICD codes	Definition
Aortic stenosis	I350	Visit a clinic or hospital for more than two with ICD code
Chronic kidney disease	N18<br>N19<br>Z49<br>Z905<br>Z94<br>Z992	eGFR<60ml/min/1.73m2 #<br><br>R3280 (kidney transplantation);<br>O7011-7020 (hemodialysis);<br>O7017; O7075 (peritoneal dialysis)<br><br>Or special codes (V codes) such as hemodialysis (V001), peritoneal dialysis (V003), or kidney transplantation (V005)
Obstructive sleep apnea	G47.30	Visit a clinic or hospital for more than two with ICD code

The estimated GFR (eGFR) was derived from the chronic kidney disease (CKD) epidemiology collaboration (CKD-EPI) equation.

Supplementary Table S2. Multivariable analysis for the incident aortic stenosis

Variables	IR	Adjusted HR	95% CI	P
Blood pressure				
Normal	2.98	Reference (1.0)		
Pre-hypertension 1	4.75	1.18	1.05-1.33	0.01
Pre-hypertension 2	4.84	1.12	1.02-1.21	0.01
Hypertension 1	9.55	1.33	1.21-1.46	<0.001
Hypertension 2	16.58	1.52	1.11-2.08	0.01
ARV				
1Q	3.46	Reference (1.0)		
2Q	3.94	1.09	0.96-1.23	0.18
3Q	4.31	1.13	1.01-1.27	0.04
4Q	5.00	1.13	1.01-1.27	0.04
5Q	8.06	1.39	1.24-1.55	<0.001
Sex				
Sex (Male)	5.21	Reference (1.0)		
Sex (Female)	4.29	0.90	0.83-0.98	0.01
Age				
Age <40y	0.74	Reference (1.0)		
40≤ & <60	5.79	6.84	6.06-7.73	<0.001
60≤ & <80	32.26	37.74	33.27-42.80	<0.001
80 ≤	140.92	178.41	120.82-263.47	<0.001
BMI				
<18.5	2.46	Reference (1.0)		
18.5≤ & <25.0	4.31	1.04	0.83-1.30	0.72
25.0≤	6.54	1.28	1.19-1.36	0.00
FBS, mg/dL				
<100	4.03	Reference (1.0)		
100≤ & <126	6.74	1.06	0.99-1.14	0.11
126≤	11.71	1.16	1.04-1.29	0.01
Cholesterol, mg/dL				
<200		Reference (1.0)		
200 ≤ & <240	1.02	1.02	0.95 - 1.09	0.59
240≤	1.05	1.05	0.96 - 1.16	0.33
Proteinuria				
<3+	4.77		Reference (1.0)	
3+≤	14.75	1.64	1.40-1.92	<0.001
Smoking				
Non smoker	5.22	Reference (1.0)		
<20pack/year	3.00	0.87	0.79-0.95	<0.001
20pack/year ≤	9.72	1.19	1.08-1.30	<0.001
CKD				
No CKD	4.71	Reference (1.0)		
History of CKD	54.43	4.23	3.58-4.98	<0.001
On dialysis	84.03	8.41	5.33-13.27	<0.001
Kidney TPL	38.17	7.38	1.84-29.54	0.01

Incidence rate: 100000 person-year

Abbreviations: BP, blood pressure; FBS, fasting blood sugar; BMI, body mass index; CKD, chronic kidney disease; TPL, transplantation; IR, incidence rate; CI, confidential interval

BP definition: normal, SBP < 120mmHg & DBP <80 mmHg; prehypertension 1, 120 mmHg ≤ SBP< 130 mmHg & DBP < 80 mmHg; prehypertension 2, 130 mmHg ≤ SBP < 140 mmHg & 80 mmHg ≤ DBP < 90

mmHg; hypertension 1, 140 mmHg ≤ SBP < 180 mmHg & 90 mmHg ≤ DBP < 120 mmHg; hypertension 2, 180 mmHg ≤ SBP & 120 mmHg ≤ DBP
